# Dr. Green and the New York Academy of Medicine

**Published:** 1859-02

**Authors:** 


*Dr. Green and the New York Academy of Medicine.*

The case of Mr. S. S. Whitney, in which death followed the application of the sponge-probang by Dr. Horace Green, excited, as might be expected, a vehement debate in the New York Academy of Medicine. A statement was made by Dr. Beales, who had charge of the patient after he left Dr. Green, to the effect that Dr. G. had erred in diagnosis, and that his death was the direct consequence of the application of the caustic by the sponge probang. It appears that after leaving Dr Green, the patient became immediately alarmingly ill, had dyspnœa, followed by extensive emphysema of the face, neck, chest and abdomen, and died one week afterward. At the *post-mortem* examination, an abscess of the size of a hen's egg was found extending a little in front of the pharynx, and downward behind and below the thyroid cartilage. It communicated with the pharynx by an opening large enough to admit the end of the forefinger. The larynx and trachea were healthy, but the mucous membrane of the bronchi was of a vermillion redness. There was recent pleurisy at the upper part of the left chest, and the upper part of the upper lobe of the left lung was hepatized. At the root of this lung, near the commencement of the bronchial ramifications, was a cavity, "about the size of a small black walnut," communicating with an opening through both pleuræ. No tubercles were found.

Dr. Beales stated that, in his opinion, the lesion of the pharynx was the immediate and direct cause of death in this case, and that the cavity found in the lung was not tuberculous. We must admit that the abscess was probably caused by the mucous membrane being accidentally lacerated by the probang. Very likely not more force was used than Dr. Green was accustomed to employ, though we believe those who have witnessed his manipulations, while they accord to him great skill and dexterity, are sometimes surprised at the boldness with which he plunges his instruments into the throat. At any rate, the accident is one which might have happened to any one, and when we consider that Dr. Green has performed the operation *over one hundred thousand times*, it is not remarkable, nor does it indicate any want of skill or care, that in one single instance he should have accidentally lacerated the mucous membrane of the pharynx.

The emphysema was undoubtedly the result of the rupture of the cavity in the left lung into the pleura, produced by the efforts of the patient in coughing; to the same cause must be traced the pneumonia and pleurisy. These complications must have concurred to produce the fatal result, and, indeed, it seems hardly probable that the patient could have died from the abscess in the pharynx alone.

It cannot be pretended that the injection of nitrate of silver into the bronchi had anything to do with the death of Mr. Whitney. The operation was only performed once, on the 6th of December, and the patient continued in his usual health up to the 14th, eight days afterward. We do not undertake to say that the introduction of a tube into the trachea, through the glottis, and the injection of caustic solutions into the air-passages, are free from all danger. Time alone can determine how far this can be performed with safety or advantage. It has received the sanction of some of the most eminent physicians in Europe, among whom are M. Trousseau, of Paris, and Dr. J. Hughes

Bennet, of Edinburgh, who have repeatedly performed the operation, not only without inconvenience, but with decided benefit to the patient. Justice demands that the facts respecting this case should be made public, and that an operation which seems to promise so much in the treatment of pulmonary complaints should not be condemned, because another operation, which almost every physician constantly employs, happened accidentally to cause death. We are glad that the case was made the subject of a full discussion in the New York Academy, and that the public are put in possession of the facts by the very full report of the proceedings in the New York Times.—*Boston Med. Jour.*

